# Effects of Heated Pneumoperitoneum on Inflammation, Oxidative Stress, and Peritoneal Histology in Female Dogs That Underwent Video-Assisted Ovariohysterectomy

**DOI:** 10.1155/2021/5515559

**Published:** 2021-10-22

**Authors:** Vanessa Milech, Juliana S. de Oliveira, Michelli A. W. de Ataide, Gabriela P. Coradini, Thaís R. Mann, Vanessa Z. Sarturi, Hellen F. Hartmann, Marcella T. Linhares, Bernardo N. Antunes, Cínthia M. de Andrade, Glaucia D. Kommers, Marco A. M. Silva, Daniel C. de M. Müller, Maurício V. Brun

**Affiliations:** ^1^Graduate Program—School of Veterinary Medicine, Universidade Federal de Santa Maria, Santa Maria 97195-000, Rio Grande do Sul, Brazil; ^2^School of Veterinary Medicine, Universidade Federal de Santa Maria, Santa Maria 97195-000, Rio Grande do Sul, Brazil; ^3^School of Veterinary and Animal Science, Universidade Federal de Goiás, Goiânia 74001-970, Goiás, Brazil

## Abstract

Laparoscopic procedures require the creation of pneumoperitoneum. CO_2_, which must be cold and dry, is the standard gas used in such surgeries. The type of gas used, its temperature, and moisture may change the peritoneal surface and cause systemic and local oxidative stress. Our objective is to evaluate the influence of pneumoperitoneum heating on the occurrence of histological lesions in the peritoneum, inflammation, plasma oxidative stress, and on the mesothelial surface in patients undergoing video-assisted ovariohysterectomy. Twenty canine females were included and distributed evenly into two groups: heated CO_2_ (HG) and unheated CO_2_ (UHG). The biomarkers of inflammation and oxidative stress were evaluated before insufflation (*T*0), at 30 min (*T*1), and at 60 min (*T*2) of exposure to CO_2_. Biopsies of the peritoneal tissue for histological evaluation were performed at *T*0 and *T*2. Regarding plasma parameters, acetylcholinesterase (AChE) showed a greater activity in the HG at *T*1 (*p*=0.0268) and *T*2 (*p*=0.0423); in turn, butyrylcholinesterase (BChE) showed a greater activity at *T*2 in the HG (*p*=0.0175) compared with *T*0. Catalase activity (CAT) was different between HG times; it was higher at *T*1 (*p*=0.0253). There was a decrease in the levels of substances reactive to thiobarbituric acid (TBARS) (*p*=0.0117) and in glutathione (GSH) (*p*=0.0114) between *T*0 and *T*2 in the UHG. Regarding tissue oxidative stress, the CAT in the HG showed a greater activity at *T*2 than *T*1 (*p*=0.0150). By comparing the groups at each time, there was a difference only at *T*2 (*p*=0.0288), being greater in the HG. Regarding the activity of superoxide dismutase (SOD) in the HG, there was a difference between *T*2 in relation to *T*0 and *T*1 (*p*=0.0181); finally, there was an increase only at *T*1 (*p*=0.0287) in the UHG when comparing groups at the same time. There were no differences in the histological parameters evaluated. Our study demonstrates that the heating of CO_2_ generates a greater inflammatory response and forms reactive oxygen species (ROS) at the plasma and peritoneal levels.

## 1. Introduction

Laparoscopic surgery induces less tissue trauma because of smaller surgical incisions and minimal tissue dissections, meticulous hemostasis, use of microsurgical instruments, and a smaller operative field [1, 2]. Such surgeries require the creation of pneumoperitoneum, which is obtained through peritoneal insufflation using dry carbon dioxide (CO_2_) (0.5% relative moisture) at 20–25°C, currently considered the gas choice for laparoscopy [3, 4].

The production of reactive oxygen species (ROS) is an integral part of metabolism and can be observed in several physiological conditions [5]. However, the excessive production of radicals may lead to oxidative stress, which arises from an imbalance between oxidant and antioxidant compounds [6]. Sammour et al. [7] argued that the surgical response associated with oxidative stress may be related to exposure of the peritoneal cavity to a nonphysiological environment, that is, ambient air in conventional surgeries and cold and dry CO_2_ in laparoscopic surgeries. In addition to such conditions, according to Brokelman et al. [8], while the minimally invasive character of laparoscopic surgery can reduce surgical trauma to the peritoneum, laparoscopic procedures introduce new elements in the abdominal cavity, such as increased abdominal pressure, insufflation gases, and changes in temperature and moisture, which may affect peritoneal biology and integrity.

The peritoneal surface is a layer very susceptible to damage and variable conditions, such as cold and dry CO_2_, during laparoscopic surgery [1]. Thus, this gas can not only induce peritoneal dehydration but also change the morphology of the mesothelium, destroying the microvilli and leading to prominence of cells with exposure of the basal lamina [9]. Lesions in the peritoneum at the time of surgery cause a fast release of platelets, fibrinogen, histamine, and vasoactive amines, resulting in ruptures of the peritoneal microcirculation [10]. The generation of ROS is also observed, and the inadequate removal and/or high levels of such products result in oxidative stress, which can cause severe metabolic dysfunctions and cell damage [11].

Mild oxidative stress can be beneficial, considering that it modulates signaling pathways and induces organism preconditioning to subsequent severe oxidative stress [12]. However, the chronicity of this process has relevant implications for the etiology of numerous chronic diseases [13], cardiovascular, neurodegenerative, renal, liver, and inflammatory diseases, and neoplasms [14]. Tissue healing can also be compromised. Studies have shown the role of ROS in vascular disorders, tissue damage, and inflammatory processes [15, 16].

In this scenario, the objective here is to evaluate the effects of heated pneumoperitoneum on the acute inflammatory response and the generation of ROS in a localized and systemic way, as well as changes in the histology of the peritoneum, using video-assisted laparoscopic surgery as a model for patients undergoing video-assisted ovariohysterectomy (OVH).

## 2. Materials and Methods

### 2.1. Animals

Twenty female mixed breed dogs (SRD), adults up to eight years of age, with an average body mass of 18.99 ± 3.87 kg, were sent by their tutors to the Hospital Veterinário Universitário da Universidade Federal Santa Maria (HVU-UFSM) to perform elective OVH. All patients included were considered healthy after undergoing a general clinical examination followed by laboratory tests (blood count, platelet count, and serum hepatic and renal biochemistry) and abdominal ultrasonography (US) to assess the genital tract. The patients were hospitalized on the day of the surgical procedure after 12 hours of preoperative fasting of solids and six hours of liquids.

The animals were randomly assigned into two groups (ten females in each group): the first group was submitted to pneumoperitoneum with unheated CO_2_ (UHG) at room temperature, and the second group was submitted to pneumoperitoneum with heated CO_2_ (HG) at 37°C.

This study was approved by the Ethics Committee on Animal Use in Education and Research (CEUA) under the protocol no. 3883261216.

### 2.2. Anesthetic Procedure

For preanesthetic medication (PAM), tramadol hydrochloride (4 mg/kg, intramuscular) was used (tramadol hydrochloride 100 mg/2 mL®; Teuto, Brazil). Fifteen minutes after PAM, a trichotomy of the operative field and the venous access site was performed with a lactated ringer to maintain fluid therapy (Sanobiol 500 mL®, Brazil). Anesthetic induction was performed with propofol (4 mg/kg, intravenous) (Diprivan 1%®; Cristália, Brazil), and the anesthetic maintenance used isoflurane (1.5 CAM) (Isoforine®, Cristália, Brazil), vaporized in 100% oxygen. Prior to the surgery, cephalothin-based antimicrobial chemoprophylaxis (30 mg/kg, intravenous) was performed (Cephalothin sodium 1 g®; ABL, Brazil). The temperature of the animals was monitored constantly, and throughout the surgical procedure, the temperature of the operating room was kept at 25–27°C.

### 2.3. Surgical Procedure

**Figure 1:**
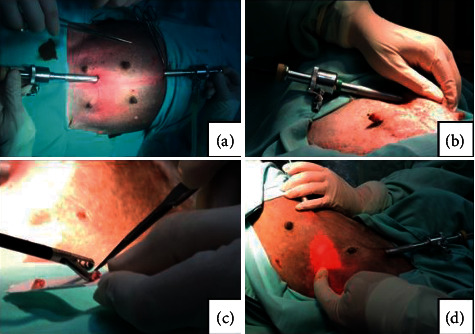
(a) Positioning of the portals for the video-assisted ovariohysterectomy technique in experimental animals. (b) Moment of peritoneal biopsy for peritoneal histology and assessment of oxidative stress at *T*0. Note that the surgeon's hand externally supports the muscle wall for collection. (c) Peritoneal tissue samples after biopsy. (d) Moment of peritoneal biopsy for peritoneal histology at *T*2 and assessment of oxidative stress at *T*1 and *T*2.

OVH was performed using the video-assisted technique with two portals, according to Tavares et al. [17] (Figure 1)(a). The first portal (11 mm) was inserted in the region of the umbilical scar using the open technique, and the second portal (11 mm), with optic visualization, was introduced in the prepubic region at a strategic point for the exteriorization of the reproductive tract. A pneumoperitoneum pressure of 10 mmHg and a constant inflation rate of 2 L/min were maintained using unheated gas (UHG) through an electronic Endoflator (Karl Storz Endoskope, Germany) at room temperature or heated gas (HG) using a Thermoflator insufflator (Karl Storz Endoskope, Germany) with its system programmed to heat the gas to a temperature of 37°C. In the case of HG, the gas was not moistened due to unavailability of such tool in the device used.

After the start of insufflation, the patients were placed in a right lateral decubitus position, making it possible to visualize the ovarian suspensory ligament and the left ovary. They were suspended using Kelly forceps. The ovary was temporarily fixed on the abdominal wall through a transparietal suture with a 4 cm curved triangular needle. Hemostasis and section of the ovarian and mesovarian vessels were performed using bipolar forceps with a cutting blade. After obtaining a section of the mesovarium and the suspensory ligament, the transparietal suture was removed from the abdominal wall, releasing the ovary; the same maneuvers were repeated on the right side with the bitches in the left lateral position. The ovaries and uterus were exposed through the wound of the second portal, allowing the application of the three cranial forceps at the cervix; the section was performed between the first and the second forceps by applying two transfixing sutures with a 2-0 thread (Polyglactin 910 2-0®; Shalon, Brazil) next to the cervix. The uterine stump was returned to the abdominal cavity under direct visualization, and insufflation was kept for 60 min, which is justified by the times of the final collections. The access wounds were sutured in three planes using a cross mattress suture with polyglactin 910 2-0 (Shalon, Brazil) in the muscle layer followed by the same suture pattern with a 3-0 thread (Polyglactin 910 3-0®; Shalon, Brazil) in the subcutaneous tissue and in the skin in an interrupted horizontal mattress pattern with 4-0 monofilament nylon (Nylon 4-0®; Technofio, Brazil).

### 2.4. Postoperative

Postoperative analgesia was performed using meloxicam (0.1 mg/kg) (Maxicam 0.2%®; Ourofino, Brazil) and an association of sodium dipyrone (25 mg/kg) with N-butylscopolamine (0.2 mg/kg) (Buscofin Compound®; Agener União, Brazil), for three days, obeying 24 h and 8 h intervals, respectively. The patients were discharged from the hospital on the same day of the surgical procedure, being referred to their tutors with recommendations for postsurgical care.

### 2.5. Collection of Samples

For analysis of inflammation, systemic and mesothelial surface oxidative stress, and histological lesions in the peritoneum, blood samples and peritoneal biopsies were collected concomitantly with the OVH surgical procedure at the stipulated times.

The assessments of inflammatory mediators and systemic oxidative stress were performed by blood biomarker analyses. For this purpose, 5 mL of blood was collected by jugular venipuncture and distributed into tubes with and without EDTA and with sodium citrate. The activities of acetylcholinesterase (AChE), butyrylcholinesterase (BChE), myeloperoxidase (MPO), thiobarbituric acid reactive substances (TBARS), reduced glutathione (GSH), catalase (CAT), superoxide dismutase (SOD), reactive oxygen species (ROS), and total thiols were analyzed. The collection times corresponded to time 0 (*T*0) (immediately after the beginning of the surgical procedure before insufflation with CO_2_), time 1 (*T*1) (30 min after exposure to CO_2_ counted from the moment when a pressure of 10 mmHg was reached), and time 2 (*T*2) (60 min after the beginning of the application of CO_2_ regardless of the completion of the surgical procedure).

For histological analysis of the peritoneum, a sample approximately 0.5 cm wide × 0.5 cm long was collected from the left cranial abdominal quadrant (*T*0) (Figure 1)(b) and the right abdominal cranial quadrant (*T*2) (Figure 1)(d). For this, the forceps were used for biopsy of video surgery, repeating the order of these collections for each patient. At the same time, for the evaluation of peritoneal oxidative stress, three more samples were collected, approximately 0.5 cm wide × 0.5 cm long, with the same instruments mentioned above. However, in this case, they were collected at *T*0, *T*1, and *T*2; those at *T*1 were also removed from the right cranial abdominal quadrant (Figure 1)(d). In this last evaluation, SOD, CAT, and TBARS were analyzed.

### 2.6. Analysis of Inflammatory Biomarkers and Plasma Oxidative Stress

The AChE activity in whole blood was determined by the method of Ellman et al. [18], modified by Worek et al. [19], while serum BChE activity was determined by the method of Ellman et al. [18] using 100 *μ*L of whole blood and 200 *μ*L of serum, respectively.

The MPO activity was measured in plasma in 200 *μ*L of whole blood collected with EDTA followed by the analysis described by Metcalf [20]. The TBARS levels were determined according to Jentzsch et al. [21] considering the measurement of the concentration of malondialdehyde (MDA) as a product of lipid peroxidation by means of reaction with thiobarbituric acid (TBA) using 600 *μ*L of serum. The reduced glutathione (GSH) was measured using a spectrophotometer with Ellman's reagent [22] using 400 *μ*L of serum for each analysis. Using 200 *μ*L of whole blood collected with sodium citrate, the CAT activity was measured by the method of Nelson and Kiesow [23]. The SOD activity was analyzed following the protocol of Mccord and Fridovich [24] using 200 *μ*L of whole blood collected with citrate. The fluorescence assay with 2′-7′-dichlorofluorescein was used to measure the production of cellular peroxide and other reactive species (ROS) [25] using a volume of 200 *μ*L of serum. The total thiol groups were analyzed using a spectrophotometer and the Ellman method [22].

### 2.7. Histological Analysis

The samples (Figure 1)(c) were fixed in 10% neutral buffered formalin, embedded in paraffin, cleaved, and stained with hematoxylin and eosin on a slide for further evaluation using light optical microscopy. The evaluation was carried out by two independent pathologists, who were unaware of the animals' origin in each group. On the peritoneal surface, the presence of congestion, hemorrhage, edema, and inflammatory cells were evaluated. The aforementioned histological changes were evaluated according to the methodology adapted from Papparella et al. [26]; scores were assigned to each lesion: 0 indicates no changes, 1 indicates mild changes (less than 10%), 2 indicates moderate changes (between 10 and 50%), and 3 indicates marked changes (more than 50%).

### 2.8. Analysis of Peritoneal Oxidative Stress

The SOD activity was carried out according to Mccord and Fridovich [24]. The assay was performed in a total volume of 1 mL containing 50 mmol of glycine buffer (pH 10), 60 mmol of epinephrine, and the aliquot of peritoneum homogenate S1 previously homogenized in phosphate buffer (1 : 5) centrifuged at 22,000 rpm for 20 min. Epinephrine was added, forming adrenochrome recorded at 480 nm using an ultraviolet-visible spectrophotometer (UV-VIS), for four min. The results are presented here in SOD IU/mg protein.

The CAT activity was measured using the method of Nelson and Kiesow [23]. For the assay, aliquots of S1 peritoneum homogenate previously homogenized in phosphate buffer (1 : 5) centrifuged at 22,000 rpm for 20 min were added to a reaction mixture containing 50 mM potassium phosphate buffer (pH 7.4), H_2_O_2_ at 10 mM, and 20 *μ*L of the sample. The reaction rate of H_2_O_2_ was monitored at 240 nm for two minutes at room temperature. The enzymatic activity was expressed in nmol/mg/protein/min.

TBARS levels in samples of peritoneal biopsy were determined according to the method described by Ohkawa et al. [27] considering the measurement of MDA concentration as a product of lipid peroxidation by means of reaction with TBA, expressed in nmol of MDA/mg of protein.

### 2.9. Statistical Analysis

The data of plasma biomarkers were submitted to the Shapiro–Wilk test to verify normality followed by a *t*-test for comparison between groups at a same time and a two-way ANOVA test of paired measurements to compare the times of a same group followed by Tukey's *post hoc* test.

To evaluate histological parameters (congestion, hemorrhage, edema, and inflammatory cells), the Shapiro–Wilk normality test was applied, and later the different moments within the groups were compared using the Wilcoxon test for paired measures. For comparison between groups at each time in isolation, the Mann–Whitney test was used. Data for variables with nonparametric distribution were expressed as median (minimum–maximum value).

The data on tissue oxidative stress for comparisons within groups between times were analyzed using one-way ANOVA for repeated samples and the Bonferroni *post hoc* test for comparison in pairs. In the comparison between groups in each time in isolation, the unpaired *t* (two-tailed) test was used. All analyses were performed using the software GraphPad Prism 6.01, and the differences were considered significant when *p* ≤ 0.05.

## 3. Results

**Figure 2:**
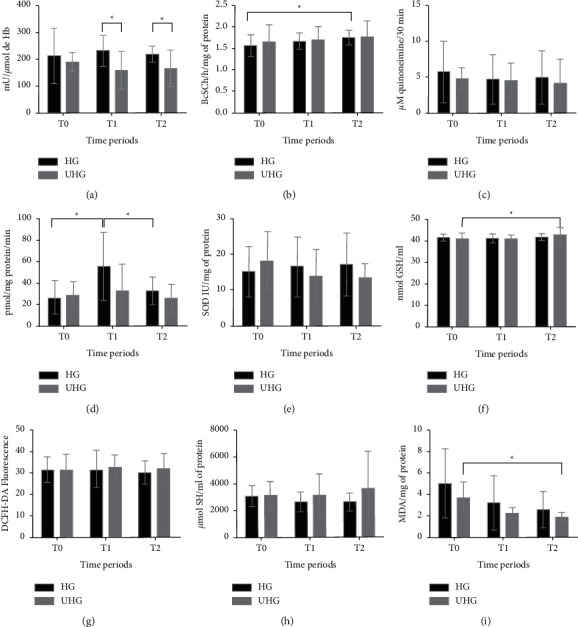
Result of inflammatory plasma biomarkers and oxidative stress. ∗ shows significant differences (*p* < 0.05) between groups or between times. T0: evaluation before insufflation with CO_2_; T1: 30 min after exposure to CO_2_ counted from the moment when a pressure of 10 mmHg was reached; T2: 60 min after the beginning of the application of CO_2_. HG = heated group, UHG = unheated group. (a) Acetylcholinesterase. (b) Butyrylcholinesterase. (c) Myeloperoxidase. (d) CAT. (e) SOD. (f) GSH. (g) Reactive oxygen species. (h) Total thiols. (i) TBARS.

The results of inflammatory biomarkers and plasma oxidative stress are shown in Figure 2. AChE and BChE were measured as indicators of low-grade systemic inflammation [28], resulting in a greater AChE activity in the HG at *T*1 (*p*=0.0268) and *T*2 (*p*=0.0423). BChE showed a higher activity at *T*2 compared with *T*0 in the HG (*p*=0.0175), however without difference in times between groups. The MPO did not indicate a significant difference between groups and times studied.

As a response to oxidative stress, SOD and CAT, which are enzymes that make up the enzymatic antioxidant defense system together with glutathione peroxidase (GPx), were considered the first line of antioxidant defense. They act in mechanisms preventing and controlling the formation of reactive species [6]. Although there were no differences in SOD levels between times and groups, there was a difference in CAT activity between *T*0 and *T*1 and between *T*1 and *T*2 in the HG (*p*=0.0253); the highest enzyme activity was at *T*1.

Still in relation to oxidative stress, the GSH activity was measured. It is considered the main cofactor of the GPx enzyme [5], which was significantly higher at *T*2 than at *T*0 in the UHG (*p*=0.0114). ROS, as well as plasma thiol concentrations, did not show any difference between the HG and UHG in any time. TBARS, which measure aldehydes, substances that stand out as secondary metabolites of lipid oxidation, were analyzed. MDA is considered the most abundant aldehyde and the main biomarker of lipid peroxidation [5, 6]. There were lower levels of TBARS at *T*2 in the UHG comparing the times *T*0 and *T*2 (*p*=0.0117).

Changes related to structural changes in the peritoneal surface, cell damage, and inflammatory response must be evaluated in histology [26, 29]. Therefore, the variables congestion, hemorrhage, edema, and intensity of the inflammatory infiltrate were measured and showed no differences within the times of a same group and when comparing the different times between groups (Table 1).

After systemic analysis, the peritoneal tissue was evaluated for local oxidative stress. The CAT activity in the HG showed a difference (*p*=0.0150) between times, with less activity at *T*1 compared with *T*2. In the UHG, there was no difference between times. Comparing the groups at each time in isolation, there was a difference at *T*2 (*p*=0.0288), at which the UHG showed lower values (29.55 ± 19.44) than HG (89.15 ± 71.28) (Table 2). Regarding the SOD activity (Table 2) in the HG, there was a difference (*p*=0.0181) between times. There was a significant increase at *T*2 in relation to baseline and *T*1. The *T*1 was not different from the baseline. In the UHG, there was no difference between times. Comparing the groups at each time in isolation, there was a difference only at *T*1 (*p*=0.0287), in which the HG showed lower values than UHG. As shown in Table 2, in the TBARS assessment, there was no difference between times and groups evaluated.

## 4. Discussion

The canine model was used considering that this study was conducted concurrently with a routine surgical procedure (OVH). Thus, the evaluation was only extended to a maximum time of 60 min based on the limits of the proposed surgery. We decided to perform the OVH considering that it is the most common surgical procedure in small animal veterinary series. It is indicated for elective sterilization and for the treatment of reproductive tract disorders [30, 31]. With this model, we sought to relate one of the routine indications for laparoscopy in small animals with inflammatory changes, generation of ROS during operation, and changes in peritoneal histology, which could be caused by heated pneumoperitoneum compared with unheated pneumoperitoneum.

Biochemical markers of oxidative stress and inflammation can detect early intracellular changes before morphological changes occur [32]. Then, oxidative and inflammatory markers were evaluated to ascertain a correlation between them and possible histological changes through the histopathological examination of the peritoneum. Considering that the cell structure of the peritoneum shows an intense inflammatory activity, with the release of inflammatory mediators, prostaglandins, interleukins, ROS, and extracellular matrix molecules, in addition to the fact that the peritoneum is a metabolically active tissue and a substrate for oxidative stress [10, 33], we believe that this would be the ideal tissue to be analyzed in this study.

Inflammation is one of the most relevant defense mechanisms. At the same time, it is one of the most common means by which tissues are damaged [34]. It can cause changes in permeability, the coagulation system, hypermetabolic response, and immunodepression and can impair intestinal function [35]. In this context, acetylcholine (Ach) has an important inflammatory suppressive action. However, it is rapidly hydrolyzed by AChE and BChE. Thus, an increase in their activity may lead to a decrease in Ach levels, thus reducing the anti-inflammatory effects [28].

In view of the results, we believe that the increased activity of AChE and BChE is related to exposure to heated and dry gas, indicating an increase in the inflammatory response of HG compared with UHG. The findings of the present study suggest that the generation of inflammation in the HG is related to the fact that low temperatures have a reducing effect on tissue metabolism [36]. In addition, it has been observed in previous studies that low temperatures reduce apoptosis, mitochondrial failure, inflammatory reactions, and the release of proinflammatory cytokines [37]. There was agreement with the study conducted by Sammour et al. [38], who found that the heating and humidification of CO_2_ did not reduce the inflammatory response.

By comparing the activity of MPO with the enzymes AChE and BChE, it can be suggested that they were more sensitive than MPO in relation to the detection of an early systemic inflammation in the evaluated groups. Inflammation and oxidative stress are closely related pathophysiological processes [39]. The MPO assesses the neutrophil accumulation index in the injured tissue [32] as these neutrophils use this enzyme to catalyze the reaction of hydrogen peroxide with chloride ions, producing the hypochlorite ion as an antiseptic [40]. Thus, it is involved in the destruction of pathogens, inflammatory conditions, and oxidative stress [41].

Oxidative stress may be a result of a deficiency in antioxidant compounds, a decrease in the activity of antioxidant enzymes (SOD, CAT, GPx) or even an increased formation of ROS [42]. By analyzing the enzymatic activity of SOD in blood in both groups evaluated, we suggest that this enzyme was not responding positively to the increase in the generation of ROS. The determination of SOD tissue activity showed conflicting results when comparing blood dosages since it was evidenced that at 30 minutes of exposure to gas (*T*1), the UHG showed higher values than HG. Such condition can be caused by the increase in energy demand with the humidification and temperature of the gas since the dry and cold gas insufflated into the hot abdomen is humidified and heated until reaching a balance with the peritoneum, thus losing temperature and liquid to achieve this situation, consuming more energy, and inducing lower temperatures [1]. Even so, considering that SOD is responsible for catalyzing the reaction of superoxide into hydrogen peroxide, considered one of the main reactions of the enzymatic antioxidant system against the superoxide radical [43], we can assume that it was effective in its function since there was no increase in tissue CAT in a same group and time evaluated.

Also evaluating SOD activity in the peritoneum, we found in the HG that there was an increase during *T*2 compared with *T*0 and *T*1. There was also an increase in CAT activity in a same group at *T*2 in relation to *T*1, and still, comparing the groups, there was an increase at *T*2 in the HG. Such results may demonstrate that there was a higher generation of ROS in the HG at 60 min of exposure to heated gas. The SOD activity was insufficient, and the CAT increased its activity to reduce hydrogen peroxide in a complementary way. Although there may be a greater expenditure of energy when the peritoneum is subjected to a cold, dry gas in an attempt to humidify and heat the medium, it must be taken into account that during exposure to cold, organisms generally regulate their metabolism positively [37]. Thus, the amount of ROS generated can be reduced in relation to the environment exposed to the heated gas, considering that the increase in metabolism may increase the production of ROS long before the induction of antioxidant protection and repair mechanisms [42]. However, it is not possible to predict for how long this response tendency continues after exposure to unheated gas since the assessment time limit was 60 min.

Furthermore, the increase in blood CAT activity in the HG from *T*0 to the end of exposure to CO_2_ is marked, thus explaining that ROS were produced in patients exposed to heated gas, considering that CAT increases its activity to reduce hydrogen peroxide. In part, the CAT activity is related to SOD because the enzymatic activity in oxidative events and cell protection depends on the amount of hydrogen peroxide that reaches the mitochondria, on the speed with which these radicals are generated, and on the antioxidant reserves [44], highlighting the secondary and complementary effects of CAT in reducing ROS.

It is also important to note that CAT activity is effective mainly at relatively high concentrations of hydrogen peroxide. For this reason, enzymes are considered indispensable for the removal of ROS, particularly in severe stress conditions [45]. The plasma membrane is permeable to hydrogen peroxide but not to the superoxide radical, and the diffusion of these compounds still occurs through aquaporins and anion channels, respectively [46]. Thus, it is believed that this, associated with a greater flow of ROS provided by the exposure of the tissue to a higher temperature [42], may result in a greater diffusion of hydrogen peroxide into the blood, increasing the plasma activity of CAT in the HG and supporting the hypothesis according to which the increase in ROS resulting from oxidative stress occurs from heat-induced cell damage.

Considered one of the main indicators of oxidative damage, GSH plays an important role in protecting cells against changes in an oxidative scenario [47]. Regarding its activity, a difference was found only in the UHG. This highlights the importance of a complete analysis of oxidative stress biomarkers and elucidates that the increase in the activity of this enzyme is an adaptive cell response to the generation of ROS and oxidative damage [48] in the UHG. Our interpretation for this result is that GSH has provided an antioxidant mechanism at the plasma level that is more effective than SOD since it did not change in the mentioned group; however, in peritoneal tissue, SOD activity was more effective against ROS.

The disagreement of these results regarding both groups analyzed in the absence of changes in the specific evaluation of ROS leads us to take into account the Lushchak exposure [43], which states that, when evaluating ROS, a range of reactive species may be included and that, at some point, may not have been detected. However, by analyzing the final product of oxidative stress or specifically antioxidants, it can be noted that ROS were generated. Our result also agrees with the findings of Rosenfeldt et al. [49], who mentioned the fact that ROS concentrations are generally low, a condition that happens because they are quickly neutralized and eliminated by the antioxidant system, even though they are highly reactive with other molecules. Thus, the activity of ROS is usually evaluated indirectly through stable metabolites or by the final products that derive from the interaction of these radicals with lipids, amino acids, or DNA.

The dose of total thiol groups is used as a parameter to measure oxidative damage to plasma proteins [50], so that the products generated during oxidative stress cause inactivation of the protein function, reducing the levels of thiols in plasma [51]. Therefore, a decrease in the levels of thiols would be observed if there was a greater oxidative manifestation, which would be revealed not only in proteins but also in lipids. Moran et al. [51] stated that proteins with access to the thiol group are in a very reducing environment, i.e., protected or buffered against oxidation. Therefore, only proteins with accessible thiol groups are involved in signaling mechanisms. This justifies our results and indicates that supposedly the generation of ROS in the UHG was attenuated by the action of blood GSH, while the HG showed detoxification of ROS through CAT's enzymatic blood activity.

The lipid peroxidation that reflects cell damage, characterized by the increase in the concentration of MDA, is a result of the involvement of ROS on cell membranes, causing the release of organelle contents and formation of cytotoxic products that may culminate in cell death [52]. However, as described by Damasceno et al. [53] and Srivastava and Kumar [42], and applied to our results regarding blood TBARS levels, transient changes in antioxidant activity may occur in response to constant exposure to ROS. This is evidenced initially by a decrease in their levels, followed or not by a compensatory mechanism reflected in their increase, due to the mobilization of stocks in other bodies. Based on this conjecture, we showed that the UHG had a decrease in TBARS levels at 60 min of evaluation, but the compensatory mechanism was not necessary since ROS were neutralized by the action of GSH in this group. Still, we did not observe which tissue changes could indicate additional antioxidant mobilization, demonstrating that the action of other antioxidant enzymes contributed to keep the ROS generated under control, preventing their harmful action on the membrane and the consequent plasma and peritoneal lipoperoxidation. We also consider that exposure to the cold environments ends up preventing any lesions related to reperfusion, lipid peroxidation, and leukotriene production [37].

Studies have indicated that low temperatures can interrupt the apoptotic pathway, preventing cell damage [54]. Our findings did not demonstrate this condition since there was no histological difference in the groups analyzed that could lead to cell damage because the measured changes are minimal. That said, we emphasize that the exposure time must have been a limiting factor in the appearance of lesions that could be identified histologically since the initial changes occur at a molecular level.

Still, it is important to remember that the exacerbated inflammatory response in the tissue starts relatively late after the ischemia-reperfusion process, and the destructive processes take some time to fully develop and continue for prolonged periods [37]. The evaluation time is quite variable in different studies; in them, the animals are submitted to 30 min [55], 50 min [56], and 120 min [3, 57] of exposure to pneumoperitoneum. The collection of the peritoneal sample and histological evaluation could reach up to 96 h [55] in search of changes, differently from what was performed in this study: the last sample collection was performed at 60 min. The occurrence of peritoneal lesions could still be justified by the evaluation of the tissue after the reperfusion event, a fact that was not observed in the present study, where collections were performed even when the cavity was insufflated with CO_2_.

Finally, we emphasize that blood measurements indicated, with a high sensitivity, changes that occur in organic systems as a whole and that can to some extent replace more invasive techniques, such as biopsies. However, it is worth emphasizing that these results demonstrate the difficulty and complexity of the enzymes of the antioxidant system, whose activity varies according to the tissue and the induction time.

## 5. Conclusions

The results obtained here do not support the hypothesis according to which heating the gas would produce greater benefits in relation to a lower inflammatory response or oxidative stress in patients undergoing elective OVH. On the contrary, our study demonstrates that the heating of CO_2_ can generate a greater inflammatory response and formation of ROS at the plasma and peritoneal levels. However, further studies on dogs and other species are needed to assess the real impacts of the strategy of heating gas especially in longer surgical procedures that may expose the patient to a greater risk of complications during the postoperative period.

## Figures and Tables

**Table 1:** Means (±SD) of the histological evaluation of the peritoneum in female dogs submitted to pneumoperitoneum with heated CO_2_ or not at baseline (*T*0) and 60 min (*T*2) of exposure to CO_2_.

Group	Time	Congestion	Bleeding	Edema	Inflammatory cells
HG	0	0.1 (±0.3)	0.2 (±0.4)	0.1 (±0.3)	0
UHG	0	0.2 (±0.4)	0.3 (±0.64)	0	0
HG	2	0.3 (±0.46)	0.3 (±0.64)	0	0
UHG	2	0.3 (±0.64)	0.5 (±0.81)	0.2 (±0.4)	0.2 (±0.6)

**Table 2:** Means (±SD) of the peritoneal analysis of CAT, SOD, and TBARS in female dogs submitted to pneumoperitoneum with heated or not CO_2_ at baseline (*T*0), 30 min (*T*1), and 60 min of exposure to CO_2_ (*T*2).

Variable/group	Times			
	*T*0	*T*1	*T*2	*p* ^*∗*^ (ANOVA)
*SOD*				
HG	85.64 (±74.31)^a^	87.56 (±65.53)^a^	253.19 (±164.07)^b^	0.0181
UHG	92.96 (±74.16)	246.29 (±182.25)	195.65 (±301.69)	0.3759
*p* ^*∗*^ (*t-*test)	0.8381	0.0287	0.6266	

*CAT*				
HG	39.96 (±42.90)^a^	24.52 (±16.11)^ab^	89.15 (±71.28)^ac^	0.0150
UHG	43.47 (±70.49)	62.05 (±50.42)	29.55 (±19.440)	0.1526
*p* ^*∗*^ (*t-*test)	0.9058	0.1069	0.0288	

*TBARS*				
HG	148.95 (±2.42)	149.35 (±0.87)	149.81 (±3.30)	0.7420
UHG	147.67 (±2.07)	149.57 (±2.59)	149.63 (±2.20)	0.1243
*p* ^*∗*^ (*t-*test)	0.219	0.8003	0.8918	

^*∗*^ *t-*test results for comparison between groups. Same superscript lowercase letters correspond to no differences to ANOVA for repeated measurements between times. Different superscript lowercase letters correspond to differences to ANOVA for repeated measurements between times.

## Data Availability

The main data regarding the methodology are described in the article. Any additional information can be requested from the authors.

## Abbreviations

AChE: Acetylcholinesterase
BChE: Butyrylcholinesterase
CAT: Catalase
ROS: Reactive oxygen species
HG: Heated CO_2_ group
UHG: Unheated CO_2_ group
GPx: Glutathione peroxidase
GSH: Reduced glutathione
MDA: Malondialdehyde
MPO: Myeloperoxidase
OVH: Ovariohysterectomy
SOD: Superoxide dismutase
TBARS: Substances reactive to thiobarbituric acid.
